# Mode water going round

**DOI:** 10.1093/nsr/nwaf114

**Published:** 2025-03-27

**Authors:** Shang-Ping Xie

**Affiliations:** Scripps Institution of Oceanography, University of California San Diego, USA

The heat capacity of the entire atmospheric column is equivalent to that of a 2.5-m-deep water column while the upper ocean that is involved in the seasonal cycle is 100–200 m deep. Because of the large thermal inertia, the ocean sets the pace of climate variability and change. The mechanisms that generate slow timescales vary from one climate phenomenon to another [1].

In winter, the surface cooling causes convection that reaches the top of the main permanent thermocline—a thin layer that separates the warm water that floats above a cold deep ocean. Deviations in surface water temperature from the regular seasonal cycle, called anomalies, are then sequestered below the seasonal thermocline that forms as the increased sunlight heats up the sea surface from spring to summer. The seasonal thermocline erodes as the sunlight weakens from fall onwards. The sequestered temperature anomalies below the seasonal thermocline are entrained into the deepening mixed layer, affecting the sea surface temperature (SST) and possibly the atmosphere in the ensuing winter. The re-emergence of sequestered water-temperature anomalies is a mechanism for SST to persist from one winter to the next [2].

The North Pacific Ocean from 15^o^N to 35^o^N features a basin-scale clockwise circulation called the subtropical gyre that is driven by wind shears between the mid-latitude westerlies and tropical easterly trades. On the western boundary of the subtropical gyre, the Kuroshio Current carries the warm tropical water northward. The Kuroshio Current leaves the Japan coast south of Osaka, becoming an eastward-flowing inertial jet called the Kuroshio Extension (KE). As atmospheric cold surges from the continent meet the warm KE current, deep mixed layers form on either flank (Fig. 1). A thick water mass of nearly vertically uniform temperatures of 16–18°C—known as the subtropical mode water—forms in the deep winter mixed layer (dark-blue shading in Fig. 1) and is subducted into the permanent thermocline to the south riding on the clockwise subtropical gyre.

**Figure 1.**
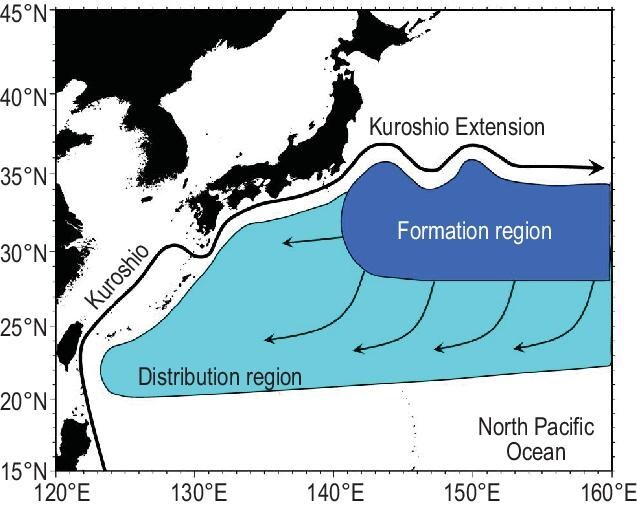
Schematic illustrating the formation and distribution of the subtropical mode water of 16–18°C. Adapted from Ref. [7].

Wu *et al.* [3] propose a re-emergence mechanism on a conveyor belt of the western subtropical gyre. Let us say that an SST anomaly forms in the winter deep mixed layer south of the KE. Subducted into the permanent thermocline, this temperature anomaly is shielded from the atmospheric damping at the surface and advected on the isopycnal (constant-density) surface. The authors detected slow propagation of temperature anomalies along the streamline on the isopycnal surface for the subtropical mode water. They suggested that the mode-water temperature anomalies eventually enter the KE region, where the winter cooling exposes the recirculating subtropical mode water to the atmosphere. The recirculation of the mode water is like a merry-go-round on the subsurface isopycnal surface, which is connected to the sea surface and the atmosphere in the deep mixed layer regions of the KE jet. This is a re-emergence mechanism not simply in a still local water column but through the mode water that is subducted 12 years earlier and returns to the KE region on the subtropical gyre merry-go-round. We call this the mode-water-go-round re-emergence mechanism.

The Atlantic Multidecadal Oscillation (AMO) and the Pacific Decadal Oscillation (PDO) are the leading modes of decadal SST variability in the Atlantic and Pacific Ocean basins, respectively. A North Atlantic warming can cause the North Pacific to warm through atmospheric teleconnection [4], e.g. advection by the atmospheric westerly flow. For a long time, the AMO and PDO have been speculated to be related to each other. Wu *et al.* [3] have added a piece to the puzzle of AMO–PDO interaction. They postulate that the mode-water-go-round re-emergence mechanism could explain the 12-year delay in SST anomalies in the broad KE region behind the AMO and that the delayed SST response to the AMO could induce the anomalous atmospheric circulation as feedback on the AMO.

Wu *et al.* [3] emphasize the advection of subtropical mode-water temperature anomalies by the background gyre circulation. The thickness of the mode water has also been observed to vary [5], affecting the upper thermocline above and the intensity of the passing tropical cyclones through the cold SST wake [6]. In the western boundary, the mode water is subject to strong dissipation and it remains to be seen how well the temperature anomalies can survive during the precarious journey into the East China Sea and off the south coast of Japan. The inertial KE jet is highly turbulent, displaying stationary and transient meanders that affect the mode-water formation [7]. Remarkably, ocean Rossby waves forced by the atmospheric Aleutian low slowly propagate westward and modulate the KE, bringing some predictability to the turbulent KE [8]. Clearly, there is more to learn about the KE and mode water as well as their change in a warming climate [9,10].

## References

[1] Xie S-P . Coupled Atmosphere-Ocean Dynamics: from El Niño to Climate Change. Amsterdam: Elsevier, 2023.

[2] Alexander MA, Deser C. J Phys Oceanogr 1995; 25: 122–37. 10.1175/1520-0485(1995)025<0122:AMFTRO>2.0.CO;2

[3] Wu B, Lin X, Yu L. Natl Sci Rev 2025; 12: nwaf047. 10.1093/nsr/nwaf047 40191249 PMC11970247

[4] Zhang R, Delworth TL. Geophys Res Lett 2007; 34: L23708. 10.1029/2007GL031601

[5] Iwasaka N, Kobashi F, Kawai Y. J Oceanogr 2024; 80: 251–72. 10.1007/s10872-024-00729-5

[6] Oka E, Sugimoto S, Kobashi F et al. Sci Adv 2023; 9: eadi2793. 10.1126/sciadv.adi2793 37703371 PMC11804680

[7] Oka E, Nishikawa H, Sugimoto S et al. J Oceanogr 2021; 77: 781–95. 10.1007/s10872-021-00608-3

[8] Nonaka M, Sasaki H, Taguchi B et al. Front Mar Sci 2020; 7: 547442. 10.3389/fmars.2020.547442

[9] Sugimoto S, Hanawa K, Watanabe T et al. Nat Clim Change 2017; 7: 656–8. 10.1038/nclimate3371

[10] Xu L, Xie S-P, Liu Q. J Geophys Res 2012; 117: C12009. 10.1029/2012JC008377

